# Early donepezil monotherapy or combination with metoprolol significantly prevents subsequent chronic heart failure in rats with reperfused myocardial infarction

**DOI:** 10.1186/s12576-022-00836-2

**Published:** 2022-06-20

**Authors:** Meihua Li, Can Zheng, Toru Kawada, Kazunori Uemura, Masashi Inagaki, Keita Saku, Masaru Sugimachi

**Affiliations:** Department of Cardiovascular Dynamics, National Cerebral and Cardiovascular Center, 6-1 Kishibe-Shimmachi, Suita, Osaka 564-8565 Japan

**Keywords:** Chronic heart failure, Donepezil, Metoprolol, Myocardial salvage, Infarct size, Reperfused myocardial infarction

## Abstract

Despite the presence of clinical guidelines recommending that β-blocker treatment be initiated early after reperfused myocardial infarction (RMI), acute myocardial infarction remains a leading cause of chronic heart failure (CHF). In this study, we compared the effects of donepezil, metoprolol, and their combination on the progression of cardiac remodeling in rats with RMI. The animals were randomly assigned to untreated (UT), donepezil-treated (DT), metoprolol-treated (MT), and a combination of donepezil and metoprolol (DMT) groups. On day 8 after surgery, compared to the UT, the DT and DMT significantly improved myocardial salvage, owing to the suppression of macrophage infiltration and apoptosis. After the 10-week treatment, the DT and DMT exhibited decreased heart rate, reduced myocardial infarct size, attenuated cardiac dysfunction, and decreased plasma levels of brain natriuretic peptide and catecholamine, thereby preventing subsequent CHF. These results suggest that donepezil monotherapy or combined therapy with β-blocker may be an alternative pharmacotherapy post-RMI.

## Introduction

Timely reperfusion via primary percutaneous coronary intervention (PCI) is the best therapeutic strategy for patients with acute myocardial infarction (AMI), and its widespread implementation has significantly reduced patient mortality [1]. Nevertheless, many individuals with AMI exhibit cardiac remodeling and chronic heart failure (CHF) due to the loss of viable myocardium, even after reperfusion therapy [2–4]. Myocardial infarct size, which is directly affected by myocardial salvage, is a strong predictor of AMI prognosis [5]. Therefore, it is necessary to rescue the myocardium to the maximum extent during the management of AMI to prevent subsequent CHF.

Sympathetic overactivity is involved in the pathophysiology of CHF. Clinical practice guidelines recommend early (< 24 h) oral administration of β-blockers, such as metoprolol [6], after coronary reperfusion in patients with AMI. Some experimental studies suggest that β-blockers decrease necrosis during myocardial infarction (MI) [7, 8], whereas others suggest that β-blockers do not have a significant effect on reperfused myocardial infarction (RMI) [9, 10]. The results of clinical studies on early β-blocker administration—mostly performed during or before the reperfusion—are controversial [11–13], and it remains to be determined whether β-blockers reduce the infarct size.

Decreased parasympathetic activity is also an independent risk factor after AMI [14, 15], indicating that parasympathetic activation can be a potential therapeutic target. Focusing on this issue, we have demonstrated that parasympathetic activation via electrical vagal nerve stimulation prevents cardiac remodeling and improves the long-term survival of rats with CHF [16]; this has resulted in several clinical studies on vagal nerve stimulation [17–19].

Recently, we proposed a novel pharmacotherapeutic approach using donepezil—central-acting acetylcholinesterase (AChE) inhibitor—to enhance the parasympathetic function in CHF. Donepezil treatment—as a monotherapy or in combination with losartan—initiated 2 weeks after permanent MI, prevented the progression of cardiac remodeling and improved the long-term prognosis in CHF rats [20, 21]. Based on the findings that early vagal nerve stimulation attenuated cardiac remodeling after RMI [22], we hypothesize that donepezil also exerts therapeutic benefits in the acute phase of RMI. This study examines the effects of donepezil, metoprolol, and their combination on myocardial salvage, infarct size, cardiac remodeling, and CHF development in a rat model of RMI.

## Methods

The care of animals and all experiments were performed in strict accordance with the Guiding Principles for the Care and Use of Animals in the Field of Physiological Science, which has been approved by the Physiological Society of Japan. All protocols were reviewed and approved (#14001, #15004, #16002, #17027) by the Animal Subject Committee of the National Cerebral and Cardiovascular Center. The experiment comprised two studies, i.e., evaluation of myocardial salvage and cardiac remodeling. During experiments, the animals were anesthetized with halothane (3% at induction, 1.2% during surgery, and 0.6% during data recording) and ventilated through an endotracheal cannula; the body temperature was maintained at 37 °C.

### RMI model

**Fig. 1:**
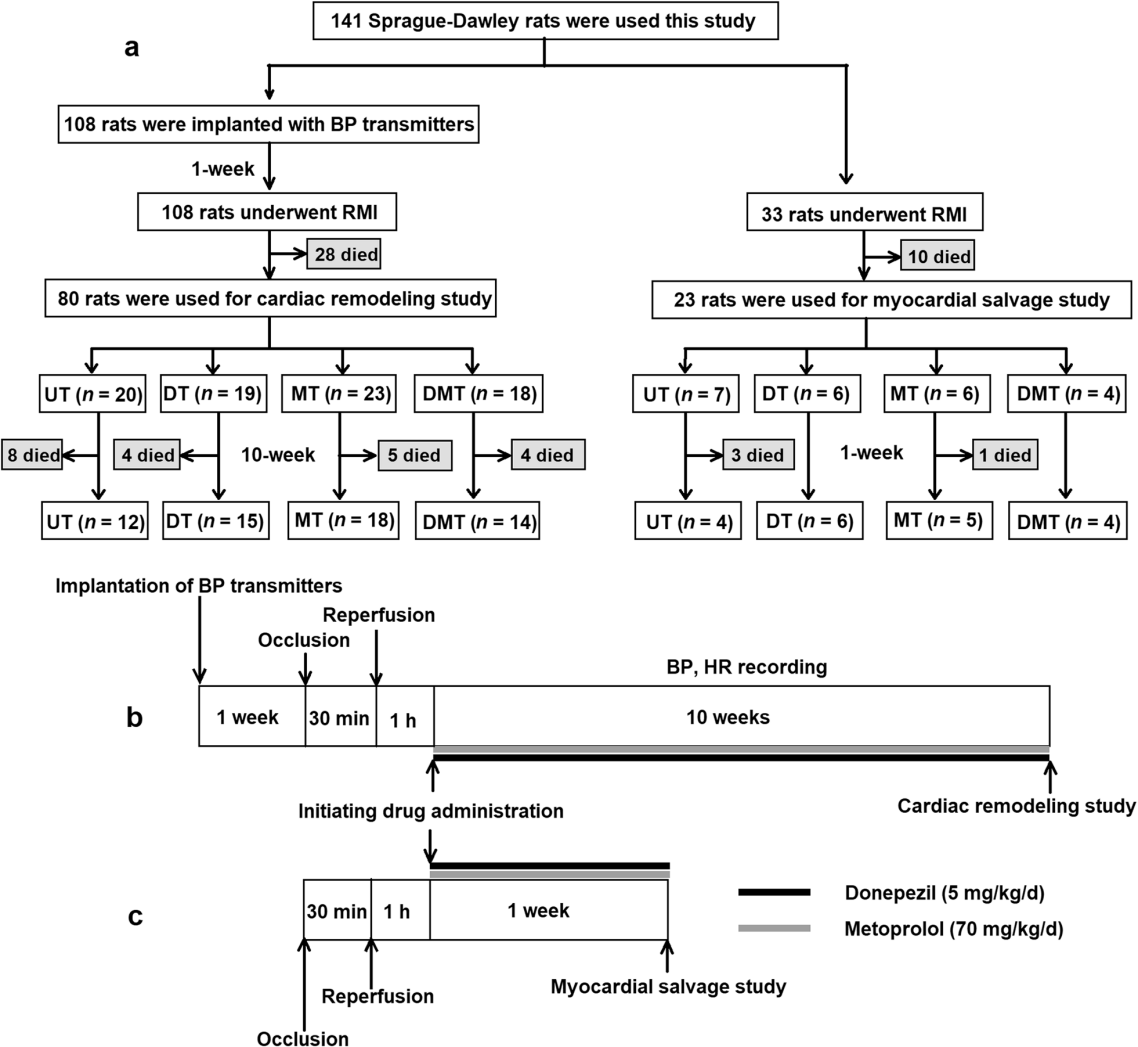
Experimental design and timeline. **a** Experimental design of UT, DT, MT, and DMT therapy in rats with chronic heart failure with reperfused myocardial infarction (RMI). **b, c** Experimental events and timelines. UT, untreated; DT, donepezil treatment; MT, metoprolol treatment; DMT, donepezil plus metoprolol treatment; BP, blood pressure; HR, heart rate

Eight-week-old male Sprague–Dawley rats (SLC, Inc., Hamamatsu, Japan) were used (*n* = 141). After inducing anesthesia, the heart was exposed, and the pericardium was opened via a left lateral thoracotomy at the second intercostal space. A 5–0 prolene suture was placed around the left ventricular proximal coronary artery (LCA) to create a reversible snare. All rats were subjected to a total of 30 min of LCA occlusion, followed by reperfusion and closing of the chest. The snare was loosened and kept in the chest to identify the ischemic risk area. Approximately 30% of the animals died during the RMI surgery due to lethal arrhythmia (Fig. 1).

### Experimental design and protocol

As shown in Fig. 1a, 80 rats that survived after RMI were used for the cardiac remodeling study, while 23 rats that survived after RMI were used for the myocardial salvage study. Rats with RMI were randomly divided into untreated (UT), donepezil-treated (DT), metoprolol-treated (MT), and donepezil plus metoprolol-treated (DMT) groups. All drugs were dissolved in the drinking water for free access after recovery from anesthesia approximately 1 h post-reperfusion. The dose of donepezil was 5 mg/kg/day (50 mg/L in drinking water), according to our previous study [20]. The dose was chosen to decrease HR by 20–30 bpm without significantly influencing weight gain in CHF rats. The dose of metoprolol was 70 mg/kg/day (700 mg/L in drinking water) according to another study in which we examined the effect of vagal nerve stimulation under β blockade [23].

### Study 1: Cardiac remodeling study

#### Conscious blood pressure (BP) and heart rate (HR) measurements

We selected a telemetry system to evaluate the non-stress hemodynamic response to drug treatments. The rats were anesthetized and ventilated through an endotracheal cannula during the implantation of the BP transmitters (TA11PA-C40; DSI, St. Paul, MN, USA). The catheter of the transmitter was inserted into the abdominal aorta. Data acquired at a 500 Hz sampling rate and an average of 10 s were recorded over a 5 min interval. One week after implantation of the BP transmitter, RMI was induced. After 10 weeks of treatment (Fig. 1b), data from the surviving rats were analyzed.

#### Hemodynamic measurements under anesthesia

Ten weeks after RMI, the hemodynamics and heart weights of survived rats with CHF were determined. Left ventricular (LV) and arterial pressures were measured using a 2-Fr catheter-tip micromanometer (SPC-320; Millar Instruments), and aortic flow was measured using a flow probe (Transonic Systems Inc.; T-206 flow probe #2.5 ss66). Right atrial pressure (RAP) was measured using a fluid-filled pressure sensor. All signals were digitized at 500 Hz for 1 min. After hemodynamic measurements, 2 mL of blood was sampled and transferred into an EDTA·2Na tube. Neurohumoral and cytokine assays were performed using the plasma obtained after centrifugation of the blood (2000 *g*, 20 min) at 4 °C.

#### Neurohumoral and cytokine assays

Plasma catecholamine concentrations were measured using high-performance liquid chromatography with electrochemical detection after alumina adsorption [20]. Plasma levels of brain natriuretic peptide (BNP) and high-sensitivity C-reactive protein (hs-CRP) were determined using enzyme-linked immunosorbent assay kits (BNP-32 Enzyme Immunoassay Kit, Peninsula Lab; Rat high-sensitive CRP ELISA, Kamiya Biochemical Company, Seattle, WA USA), according to the manufacturer’s instructions.

#### Histological assessments of risk area, infarct size, cardiac fibrosis, and myocyte cross-sectional area

Rats were euthanized with an overdose of pentobarbital sodium (100 mg/kg, iv) after hemodynamic measurements and blood sampling under anesthesia. Following re-occlusion of the LCA using the residual snare, the heart was quickly harvested and mounted on a modified [24, 25] Langendorff apparatus. The blood was flushed out using 0.9% saline, and the perfusate was infused with Pigment Blue Ink (Platinum Co., Ltd., Japan) to identify the ischemia/reperfusion (I/R) regions. Once the dye had stained the non-ischemic regions, biventricular weights were determined, and the hearts were sectioned. Three portions (1 mm thick) from the apex to the base were selected and embedded in paraffin. Sections (4 µm thick) were cut and stained with Masson’s trichrome. Histological images were obtained using a microscope. The area at risk was calculated as the percentage of the unstained I/R region relative to the whole LV area, which was averaged for the three portions. The infarcted area was calculated as the percentage of the area of scar tissue relative to the whole LV area, which was averaged for the three portions. Infarct size was defined as the percentage of the infarcted area relative to the area at risk. The extent of cardiac fibrosis was evaluated using a light microscope (BZ-9000; Keyence, Osaka, Japan) coupled with image-analysis software at 20 × magnification. The myocyte cross-sectional area (papillary cardiomyocytes) was then evaluated at 40 × magnification. The area of cardiac fibrosis in each heart was calculated as the ratio of the blue area to the total tissue area in eight fields of the noninfarcted tissue.

#### Myocardial microvessel density

Biventricular sections were generated using the method described in the histological assessments. The sections were incubated with a rabbit anti-human von Willebrand factor (vWF, 1:100 dilution, Dako, Denmark) polyclonal antibody and then with Alexa Fluor 633-conjugated goat anti-rabbit IgG antibody for analysis of microvessel density. Capillary vessels in the peri-infarct area and septum were counted as fluorescent regions using a laser-scanning microscope at 20 × magnification. Data obtained from 6 to 8 fields were expressed as the number of capillary vessels.

### Study 2: myocardial salvage study

To elucidate the therapeutic mechanism of donepezil, we designed a myocardial salvage study. After 1 week of the treatments (Fig. 1c), the hearts were harvested from the survived rats with RMI and subjected to histological and immunohistochemical analyses.

#### Myocardial neutrophil infiltration

The middle portion of the biventricular short axis of the heart was embedded in paraffin, sectioned transversely to 4 µm thickness, stained with Masson’s trichrome to confirm the I/R region, and stained with hematoxylin and eosin to analyze myocardial neutrophil infiltration. In each rat, the numbers of neutrophils were counted using a light microscope at 40 × magnification in six fields randomly selected from the I/R region.

#### Myocardial macrophage infiltration

Heart sections were prepared to examine macrophage infiltration and incubated with mouse anti-rat CD68 (1:100 dilution; Abcam, Tokyo, Japan) and rabbit anti-human vWF antibodies. The sections were then incubated with Alexa Fluor 488-conjugated goat anti-mouse IgG and Alexa Fluor 633-conjugated goat anti-rabbit IgG antibodies. The fluorescence of Alexa Fluor 488 (CD68), Alexa Fluor 633 (vWF), and Alexa Fluor 350 (nuclei) was observed using a fluorescence microscope at 40 × magnification. To distinguish between classically activated proinflammatory type 1 macrophages (M1) and alternatively activated anti-inflammatory type 2 macrophages (M2), the following were stained together: (1) CD68, CD80 (1:200 dilution; BIS, USA), and DAPI for M1; or (2) CD68, CD163 (1:200 dilution; BIS, USA), and DAPI for M2 [24]. Data obtained from six fields were averaged and expressed as the percentage area of macrophage infiltration.

#### Myocardial apoptosis

Myocardial apoptosis was analyzed in the I/R region. Apoptotic cells were detected using terminal deoxynucleotidyl transferase dUTP nick end labeling (TUNEL) staining [25]. Biventricular sections were incubated with the TUNEL reagent (ApopTag® Fluorescein In Situ Apoptosis Detection Kit, Millipore.com, USA & Canada) and rabbit anti-connexin 43 (Cox43) antibody (1:100 dilution; Sigma, Inc., MO, USA), followed by incubation with Alexa Fluor 633-conjugated goat anti-rabbit IgG antibody. The fluorescence of Alexa Fluor 488 (TUNEL), Alexa Fluor 633 (Cox 43), and Alexa Fluor 350 (nuclei) were observed under a fluorescence microscope. Apoptotic myocardial cells were counted at 20 × magnification. Data obtained from six fields were averaged and expressed as the number of apoptotic cells.

## Statistical analysis

All statistical analyses were performed using GraphPad Prism 7 (GraphPad Software, San Diego, CA, USA). All data are expressed as means ± standard errors of the means (SEMs) or box-and-whisker plots. Long-term-recorded data on HR and mean BP (MBP) before and during treatment within each group and among the groups were compared using a one-way analysis of variance (ANOVA), followed by Dunnett’s test. For data obtained from the hemodynamic and remodeling study, differences between groups were tested using one-way ANOVA, followed by Tukey’s multiple comparisons test. Data regarding the myocardial salvage study, cardiac fibrosis, infarct size, microvessel density, neurohumoral assays, and cytokine assays were analyzed using nonparametric Kruskal–Wallis tests followed by Dunn’s tests. Differences were considered significant at *P* < 0.05.

## Results

### Study 1: cardiac remodeling study

#### Conscious hemodynamic responses

**Fig. 2:**
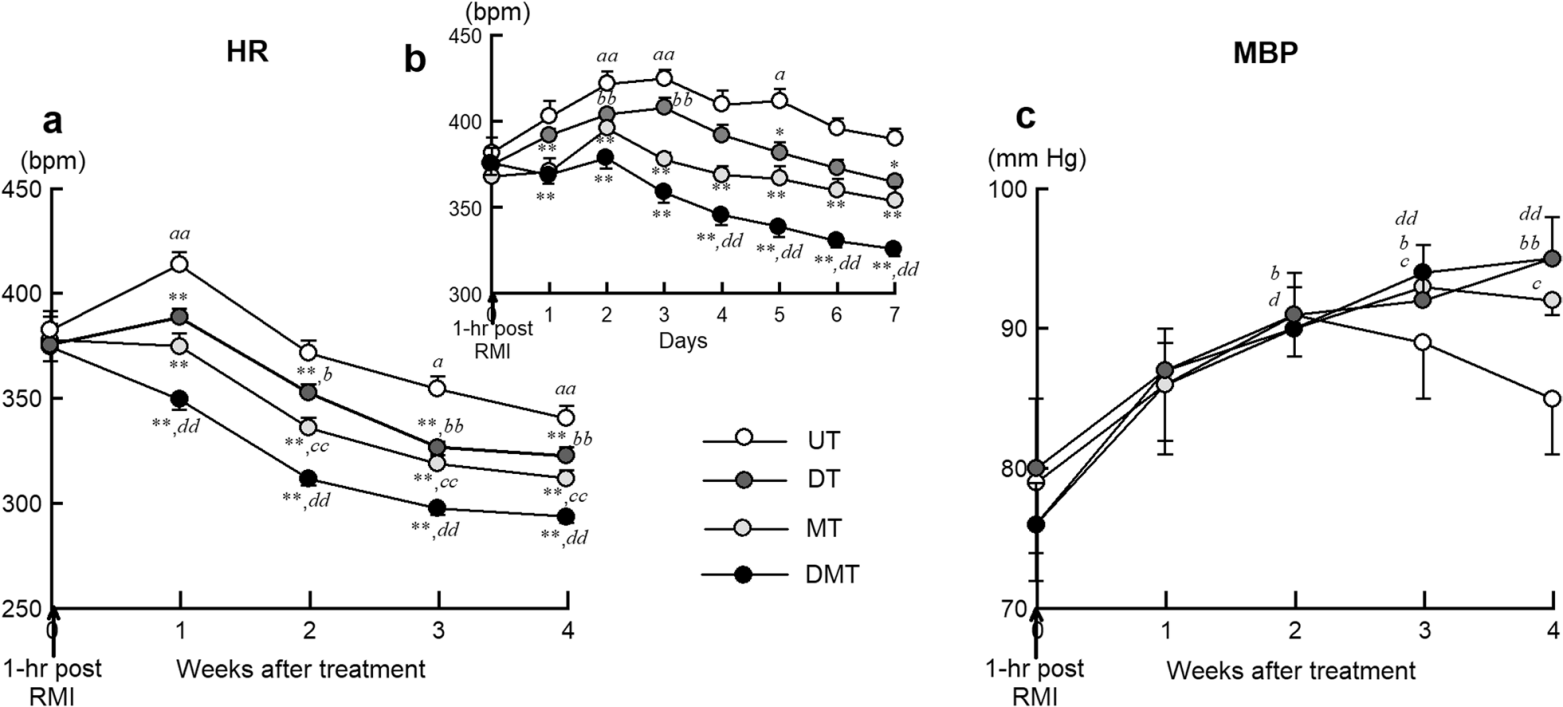
Effects of treatment on heart rate (HR) and mean blood pressure (MBP) of rats with RMI-induced CHF, recorded using telemetry. **a** Weekly HR. Each point represents averaged 1-week data for all animals in each group. **b** Daily average HR during week 1. Each point represents the averaged 1-day data for all animals in each group. (UT, *n* = 14; DT, *n* = 16; MT, *n* = 18; DMT, *n* = 14). **c** Weekly MBP. Each point represents averaged 1-week data for all animals in each group. Values are expressed as means ± SEMs. **a***–***d** *P* < 0.05; *aa, bb, cc* and *dd P* < 0.01 vs. pretreatment value (week 0, day 0, at 1-h post-RMI) within each group (UT, DT, MT or DMT); *P < 0.05, **P < 0.05 vs. UT groups, using one-way ANOVA and post-hoc Dunnett’s test. CHF, chronic heart failure

In the cardiac remodeling study, the weekly average HR in the UT group increased to 413 bpm during week 1 and then decreased (Fig. 2a). Compared with that in the UT group, the weekly average HR in the other groups was significantly reduced. This reduced HR was most prominent in the DMT group. The difference in HR between the DMT and UT groups was significant and at approximately 40 bpm during week 4 of treatment. Figure 2b shows the daily average HR during the first 7 days. The HR increased, reached a maximum (425 bpm) on day 3, and gradually decreased in the UT group. The difference in HR between the DMT and UT groups was significant and at approximately 60 bpm on day 7. Similarly, the HR in the DT and MT groups was decreased significantly compared with that in the UT group.

The weekly MBP in the UT group reached a peak during week 2 and then gradually tended to decrease. In contrast, MBP in the DT, MT, and DMP groups continuously increased from week 1 to week 4 (Fig. 2c). The difference in MBP between the DT or DMT and UT groups was approximately 10 mm Hg during week 4.

#### Hemodynamics under anesthesia

The hemodynamic, cardiac remodeling, and cardiac function parameters after 10 weeks of treatment are shown in Table 1. The body weights of the rats in the DMT group were higher than those in the UT group. Rats in the DT and DMT groups had lower heart weights, higher cardiac index (CI), lower LV end-diastolic pressure (LVEDP), higher maximum dp/dt LV pressures (LV dp/dt_max_), and minimum dp/dt LV pressures (LV dp/dt_min_) than the rats in the UT group. Compared with UT, the weekly average HR was significantly lower in DT, MT, and DMT during week 4 under the conscious conditions (Fig. 2a). In contrast, there were no significant differences in HR between UT, DT, MT, and DMT under the anesthetized conditions (Table 1), possibly because of the suppression of autonomic nervous activities.

**Table 1:** Hemodynamic, cardiac remodeling, and plasma neurohumoral and inflammatory parameters in rats with RMI-induced chronic heart failure (CHF) after 10 weeks of treatment

	UT group (*n* = 12)	DT group (*n* = 15)	MT group *n* = 18)	DMT group (*n* = 14)
BW, g	495 ± 7	523 ± 7	525 ± 10	545 ± 9^**^
HW, g/kg	2.73 ± 0.13	2.39 ± 0.05^*††^	2.73 ± 0.06	2.41 ± 0.03^*†^
Infarcted area, %	20.1 ± 1.2	12.5 ± 1.2^**††^	21.1 ± 1.1	13.9 ± 1.5^*††^
MBP, mmHg	96 ± 3	98 ± 3	96 ± 3	107 ± 3
HR, bpm	302 ± 7	312 ± 6	304 ± 4	317 ± 7
CI, mL/min/kg	98 ± 6	120 ± 4^*††^	91 ± 4	120 ± 4^*††^
LV dp/dt_max_, mmHg/s	4318 ± 127	4920 ± 151^*^	4387 ± 180	4950 ± 120^*†^
LV dp/dt_min_, mmHg/s	3493 ± 147	4120 ± 127^*†^	3511 ± 166	4277 ± 113^**††^
LVEDP, mmHg	23 ± 3	14 ± 2^*^	21 ± 2	14 ± 1^*^
RAP, mmHg	2.9 ± 0.3	3.1 ± 0.4	3.4 ± 0.3	2.6 ± 0.5
Plasma NE, pg/mL	412 ± 72	139 ± 15^*^	281 ± 51	103 ± 13^**†^
Plasma Epi, pg/mL	430 ± 77	111 ± 45^**††^	382 ± 86	119 ± 22^**†^
Plasma BNP, pg/mL	553 ± 42	370 ± 16^**††^	482 ± 20	417 ± 8^**^
Plasma hs-CRP, ng/mL	825 ± 67	362 ± 61^**^	415 ± 38^*^	201 ± 12^**††^

For data from the hemodynamic and remodeling studies under anesthesia, differences between the UT, DT, MT, and DMT groups were determined using one-way ANOVA, followed by Tukey’s multiple comparison tests. For data on the infarcted area, plasma neurohumoral, and inflammatory parameters, differences between UT, DT, MT, and DMT groups were determined using nonparametric Kruskal–Wallis tests followed by Dunn’s tests. BW: body weight; HW: biventricular weight normalized by body weight; MBP: mean arterial blood pressure; HR: heart rate; CI: cardiac index; LV dp/dt_max_: maximum dp/dt of the left ventricular pressure; LV dp/dt_min_: minimum dp/dt of the left ventricular pressure; LVEDP: left ventricular end-diastolic pressure; RAP: right atrial pressure. NE: norepinephrine; Epi: epinephrine; BNP: brain natriuretic peptide; hs-CRP: high-sensitivity C-reactive protein; UT: untreated; DT: donepezil treatment; MT: metoprolol treatment; DMT: donepezil plus metoprolol treatment; RMI: reperfused myocardial infarction

Data are shown as means ± SEMs. *P < 0.05; **P < 0.01 versus UT group, ^†^*P* < 0.05; ^††^*P* < 0.01 versus MT group.

#### Cardiac structure

**Fig. 3:**
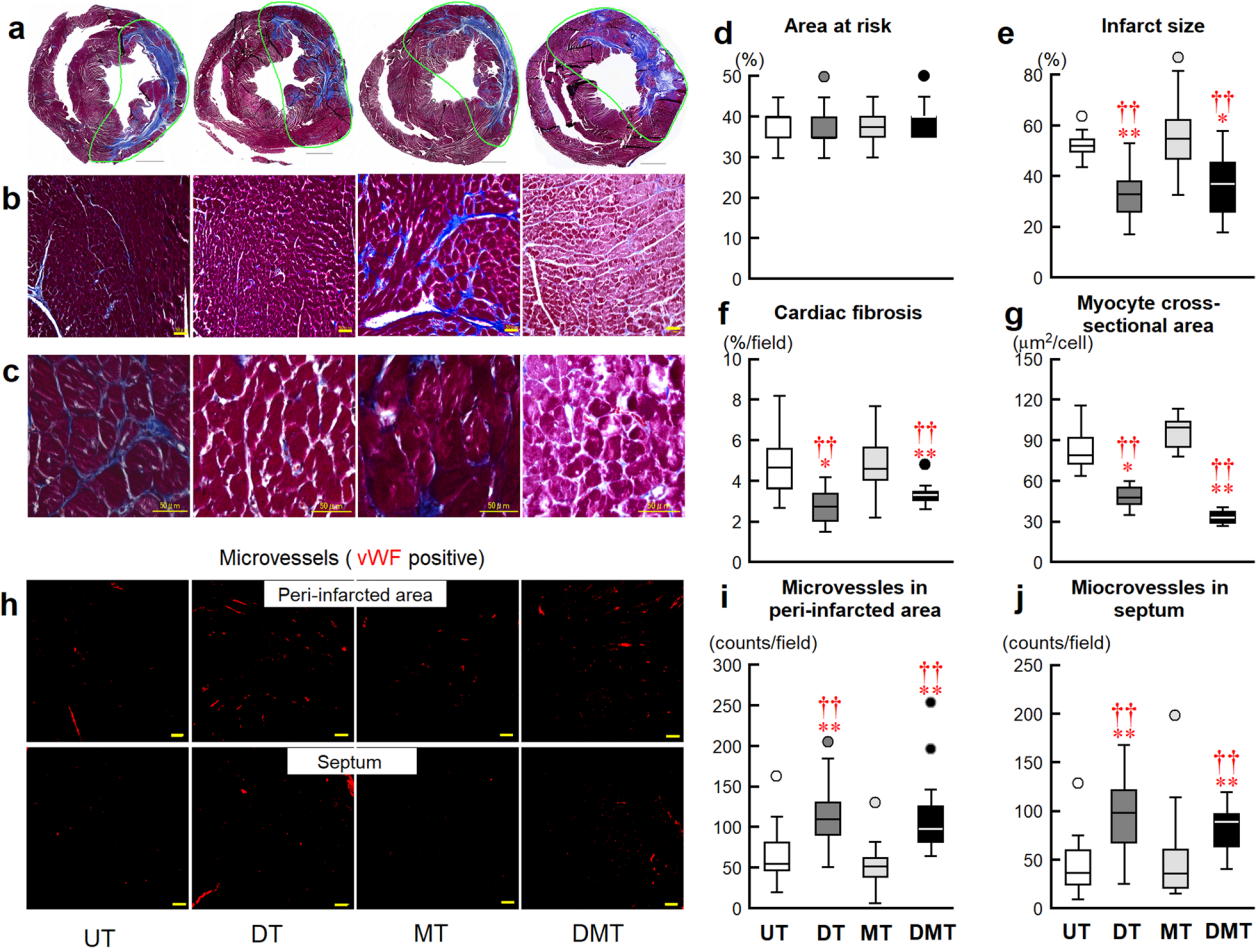
Masson’s trichrome staining of ventricles in rats with CHF 10 weeks after treatment. **a** Representative biventricular sections of RMI. The region surrounded by a green line showed area at risk. Scale bars, 2 mm. **b** Representative images of cardiac fibrosis in the noninfarcted area. **c** Representative myocyte cross sections (papillary cardiomyocytes). Scale bars, 50 μm. **d** Area at risk (UT, 38 ± 5%, *n* = 12; DT, 38 ± 6%, *n* = 15; MT, 38 ± 4%, *n* = 18; DMT, 39 ± 5%, *n* = 14). **e** Infarct size (infarcted area/area at risk) (UT, 51 ± 2%, *n* = 12; DT, 33 ± 3%, *n* = 15; MT, 56 ± 3%, *n* = 18; DMT, 35 ± 3%, *n* = 14). **f** Cardiac fibrosis index in the noninfarcted regions (UT, 5.0 ± 0.6%/field, *n* = 10, 60 fields of view; DT, 2.7 ± 0.2%/field, *n* = 14, 84 fields; MT, 4.9 ± 0.3%/field, *n* = 18, 108 fields; DMT, 2.4 ± 0.2%/field, *n* = 14, 84 fields). **g** Myocyte cross-sectional area in the noninfarcted region (UT, 1118 ± 98 μm^2^/cell, *n* = 7, 312 cells; DT, 683 ± 34 μm^2^/cell, *n* = 12, 668 cells; MT, 1218 ± 123 μm^2^/cell, *n* = 7, 264 cells; DMT, 465 ± 33 μm^2^/cell, *n* = 6, 869 cells). **h** Representative micrographs of blood microvessels (von Willebrand factor [vWF, red]) immunostaining in the peri-infarct and noninfarcted (septum) regions in rats with CHF. Scale bars, 50 μm. Quantitative analysis of microvessel density in (**i**) peri-infarct region (UT, 64 ± 10/field, *n* = 6, 21 fields of view; DT, 119 ± 11/field, *n* = 6, 21 fields; MT, 52 ± 7/field, *n* = 6, 22 fields; DMT, 103 ± 8/field, *n* = 7, 36 fields); (**j**) in the septum (UT, 44 ± 8/field, *n* = 6, 16 fields; DT, 105 ± 18/ field, *n* = 6, 11 fields; MT, 62 ± 20/field, *n* = 6, 16 fields; DMT, 82 ± 8/field, *n* = 7, 22 fields). Values are expressed as means ± SEMs. Data are shown as box-and-whisker plots for area at risk, MI size, cardiac fibrosis, myocyte cross-sectional area, and microvessels in all animals. *P < 0.05, **P < 0.01 vs. UT; ^††^*P* < 0.01 vs. MT, using nonparametric Kruskal–Wallis tests, followed by Dunn’s tests

Histological analyses revealed the association of preserved cardiac function with histological changes in rats in the DT and DMT groups (Fig. 3a–c). Rats in the DT and DMT groups exhibited a significantly reduced infarct size compared with those in the UT group. Similarly, rats in the DT and DMT groups exhibited significantly suppressed cardiac fibrosis and markedly attenuated cardiac myocyte hypertrophy as a direct consequence of reduced myocyte cross-sectional area (Fig. 3d–g). Immunohistochemical analysis of vWF in the peri-infarct region and septum revealed that neovascularization was more pronounced in the DT and DMT groups than in the UT group (Fig. 3h). Quantitative analysis confirmed that capillary density in the peri-infarct and septum regions was significantly higher in the DT and DMT groups than in the UT group (Fig. 3i, j). These beneficial effects were not observed in the MT group.

#### Neurohumoral and cytokine levels

Rats in the DT and DMT groups showed lower plasma levels of norepinephrine, epinephrine, BNP, and hs-CRP than those in the UT group. Metoprolol monotherapy reduced the plasma hs-CRP levels compared with those observed in the UT group (Table 1).

### Study 2: myocardial salvage study

**Fig. 4:**
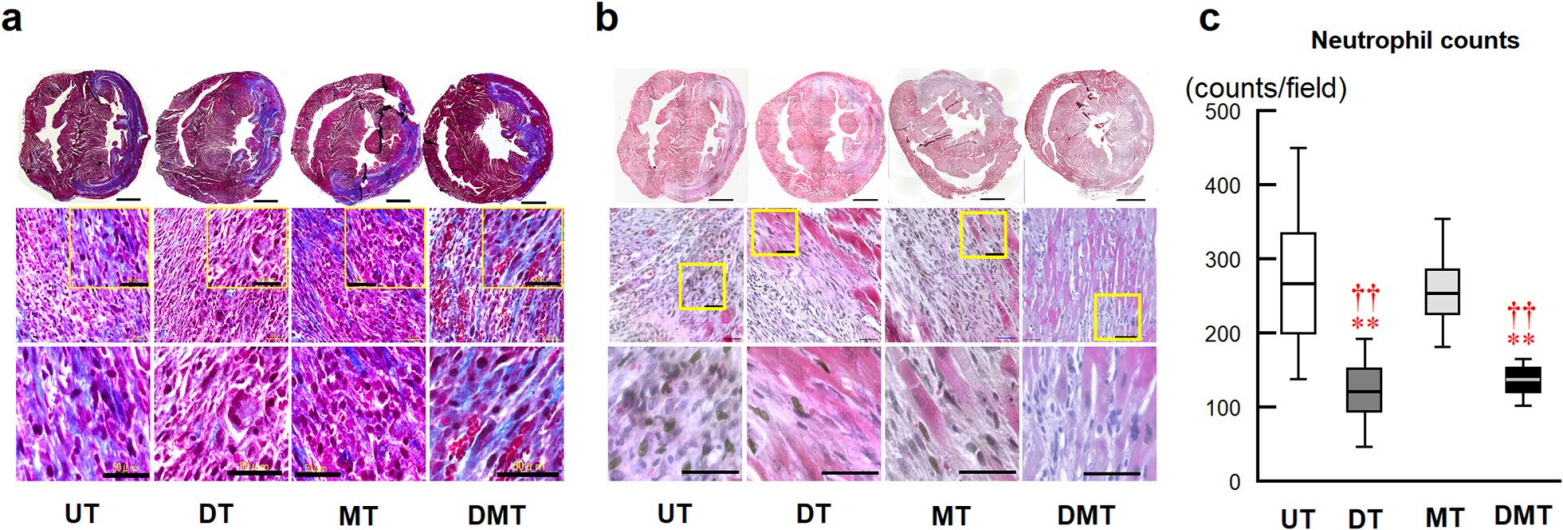
Neutrophil infiltration of rat myocardium and histological analysis on day 8 after reperfused myocardial infarction (RMI). Representative photomicrographs of Masson’s trichrome staining (**a**), and hematoxylin and eosin staining of the heart (**b**) and the ischemia/reperfusion (I/R) region. The whole heart scale bars are 2 mm. Infiltrating neutrophils in the I/R region, scale bars are 50 μm. **c** Box-and-whisker plots of neutrophil counts in the I/R region (UT: 271 ± 15/field, *n* = 5, 30 fields; DT: 123 ± 7/field, *n* = 5, 30 fields; MT: 257 ± 10/field, *n* = 4, 24 fields; DMT: 136 ± 4/field, *n* = 4, 24 fields). Data are expressed as means ± SEMs. **P < 0.01 vs. UT; ^††^*P* < 0.01 vs. MT, using nonparametric Kruskal–Wallis tests, followed by Dunn’s test

**Fig. 5:**
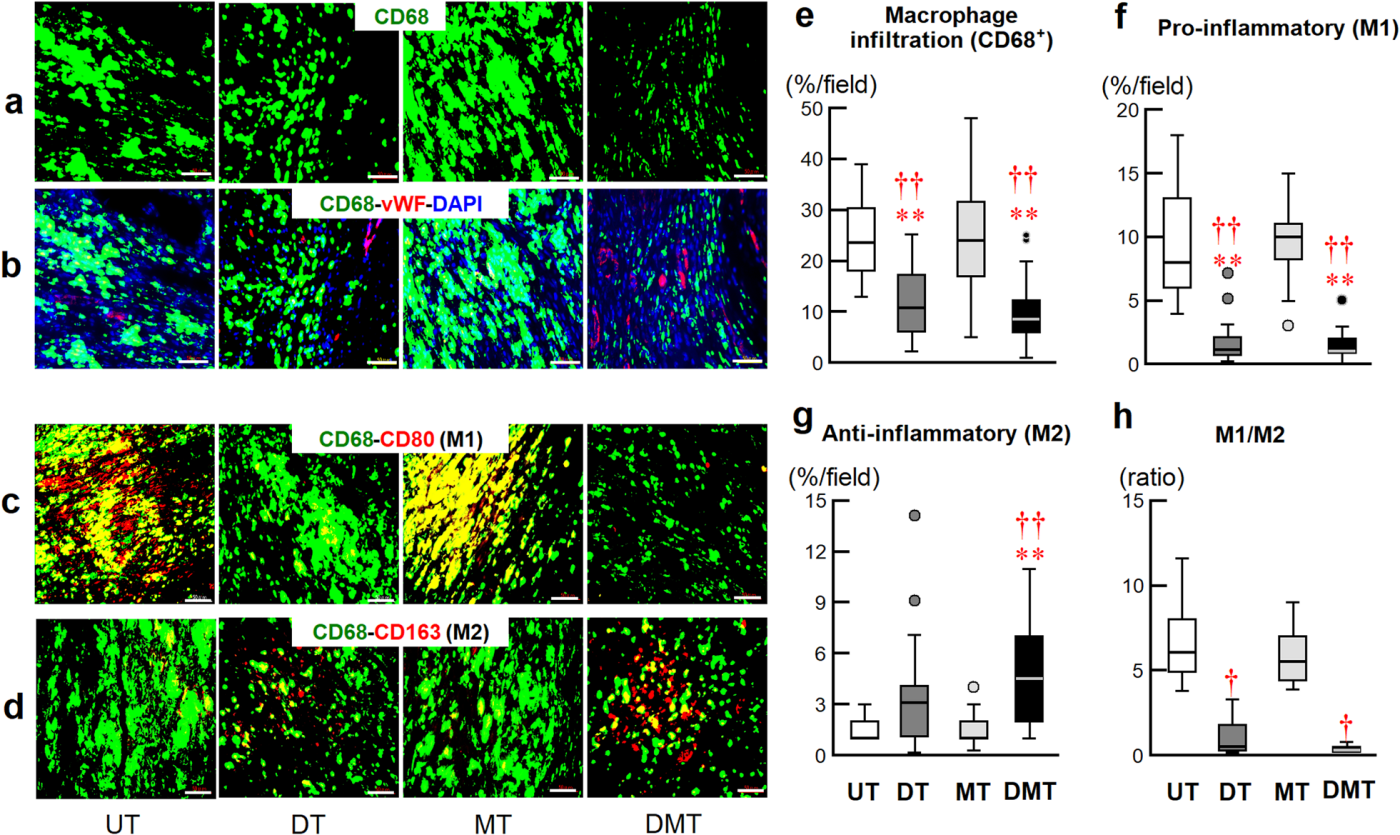
Immunohistological staining of macrophages on day 8 post-RMI. Representative micrographs of CD68 (**a**), and triple CD68, vWF, and DAPI staining of the I/R region (**b**). Representative macrographs of CD68 and CD80 cells (proinflammatory M1) (**c**), and CD68 and CD163 cells (anti-inflammatory M2) (**d**) in the I/R region. Scale bars, 50 μm. **e** Quantitative analysis of macrophage infiltration (UT:24 ± 3%/field, *n* = 4, 24 fields of view; DT: 11 ± 2%/field, *n* = 6, 36 fields; MT: 24 ± 2%/field, *n* = 5, 30 fields; DMT: 10 ± 3%/field, *n* = 4, 32 fields). **f** M1 infiltration (UT: 9.4 ± 0.8%/field, *n* = 4, 24 fields; DT: 1.6 ± 0.9%/field, *n* = 5, 30 fields; MT: 9.8 ± 0.5%/field, *n* = 5, 30 fields; DMT: 1.5 ± 0.2%/field, *n* = 4, 24 fields). **g** M2 infiltration (UT: 1.5 ± 0.1%/field, *n* = 4, 24 fields; DT: 3.1 ± 0.5%/field, *n* = 5, 30 fields; MT: 1.5 ± 0.2%/field, *n* = 5, 30 fields; DMT: 4.8 ± 0.6%/field, *n* = 4, 24 fields). **h** M1 to M2 ratio (UT: 6.9 ± 1.7, *n* = 4; DT: 1.1 ± 0.6, *n* = 5; MT: 6.0 ± 0.9, *n* = 5; DMT: 0.4 ± 0.1, *n* = 4). M1, macrophage type 1; M2, macrophage type 2. Data are expressed as means ± SEMs. **e**–**h** Box-and-whisker plots based on macrophage, M1, and M2 infiltration and the M1/M2 ratio in all fields. *P < 0.05, **P < 0.01 vs. UT; †*P* < 0.05, ^††^*P* < 0.01 vs. MT, using nonparametric Kruskal–Wallis tests, followed by Dunn’s test. I/R, ischemia/reperfusion

**Fig. 6:**
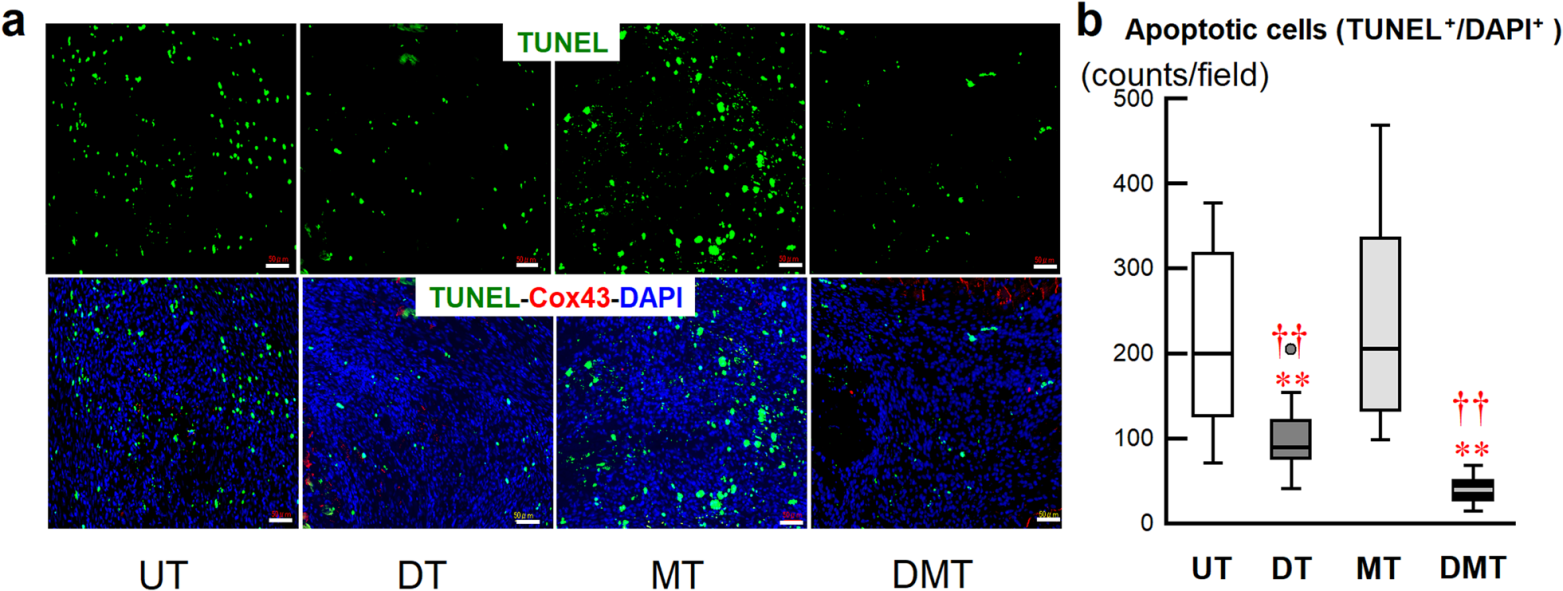
Analysis of myocardial apoptosis on day 8 post-RMI. **a** Representative micrographs of TUNEL, Cox43 (connexin 43), and DAPI staining of the I/R region. Scale bars, 50 μm. **b** Quantitative analysis of apoptotic cells (UT: 213 ± 3/field, *n* = 4, 24 fields; DT: 99 ± 8/field, *n* = 6, 36 fields; MT: 236 ± 31/field, *n* = 5, 30 fields; DMT: 39 ± 2/field, *n* = 4, 24 fields). Data are expressed as means ± SEMs. **B** Box-and-whisker plots based on apoptotic cells in all fields of each group. **P < 0.01 vs. UT; ^††^*P* < 0.01 vs. MT, using nonparametric Kruskal–Wallis tests, followed by Dunn’s test

We evaluated myocardial salvage as a function of myocardial immune cell infiltration and myocardial apoptosis on day 8 after RMI. Figure 4 shows neutrophil infiltration associated with the inflammatory response. Representative images of Masson’s trichrome staining (Fig. 4a) and hematoxylin and eosin staining (Fig. 4b) indicate the I/R region and neutrophil infiltration, respectively. Considerable neutrophil infiltration into the myocardium of RMI tissues was observed in the UT and MT groups and was significantly reduced in the DT and DMT groups (Fig. 4c). Immunohistochemical staining (Fig. 5) of CD68^ +^ cells (all macrophages, Fig. 5a), CD68^+^–vWF^ +^ cells (microvessels, Fig. 5b), CD68^+ ^–CD80^+^ cells (M1, Fig. 5c), and CD68^ +^–CD163^+^ cells (M2, Fig. 5d) in RMI tissues revealed decreased total macrophage infiltration (Fig. 5e), decreased M1 cell counts (Fig. 5f), increased M2 cell counts (Fig. 5g), and increased microvessels signals (Fig. 5b) in the I/R region in the DT and DMT groups, compared with those in the UT and MT groups, resulting in the lower M1/M2 ratio in the DT and DMT groups than in the MT group (Fig. 5h). Myocardial apoptotic cell counts were significantly lower in the DT and DMT groups than those in the UT and MT groups, and strong Cox43 signals were observed in the DT and DMT groups (Fig. 6a). Quantitative analysis demonstrated that the number of myocardial apoptotic cells was significantly lower in the DT and DMT groups than that in the UT and MT groups (Fig. 6b).

## Discussion

To the best of our knowledge, the present study demonstrates for the first time that increasing parasympathetic function early after RMI with donepezil, either as a monotherapy or a combined therapy with metoprolol, prevents the progression of CHF. The major findings are that donepezil treatment (i) significantly suppressed increases in daily HR during the acute phase, decreased weekly HR, and preserved MBP during the chronic phase; (ii) reduced infarct size by contributing to myocardial salvage through anti-inflammatory effects and reducing myocardial apoptosis in the heart during the early stages of RMI; and (iii) prevented the progression of cardiac remodeling and dysfunction by suppressing cardiac fibrosis, alleviating neurohumoral activation, and promoting cardiac angiogenesis.

### Effects of early donepezil, metoprolol, and combined donepezil–metoprolol therapy on HR and MBP in RMI-induced CHF

We used a telemetric system to monitor arterial MBP and HR in unstressed conscious rats. After RMI, HR increased and peaked (425 bpm, Fig. 2b) on day 3 in the UT group, likely caused by compensatory sympathetic activation. Either donepezil or metoprolol significantly attenuated the HR responses (from week 1), and the combination of these two drugs exerted additive effects concerning HR reduction in the RMI rats (Fig. 2a). These bradycardic effects persisted in the chronic phase (week 4). Furthermore, weekly HR in the DMT group during week 4 post-RMI was reduced to nearly that observed in sham-operated animals [16]. HR has been recognized as the best predictor of prognosis after MI in patients with CHF. Owing to their ability to induce bradycardia, β-blockers have become very important in treating patients with CHF [26]. Metoprolol and carvedilol are the 2 most common β-blockers prescribed following MI. Metoprolol is a primarily β_1_-adrenergic receptor blocker, while carvedilol is a β_1_, β_2_, and α_1_-adrenergic receptor blocker which also has pleiotropic anti-oxidant and vasodilatory effects [27]. Despite the differences in the pharmacological effects, however, none of the clinical studies done so far was conclusive enough to recommend one over the other as a guideline for after MI management [27]. To address the impact of HR reduction after MI and simplify the pharmacological interpretation, we selected metoprolol in the present study. The results showed that metoprolol exerted a bradycardic effect but did not significantly improve cardiac function in this rat model of RMI.

β-Adrenergic receptors are expressed on sinus pacemaker cells and cardiac myocytes, and thus, peripheral β-blockers not only decrease HR but also suppress myocardial contractility. These negative inotropic effects may limit the compensatory mechanism for the maintenance of cardiac output in CHF subjects whose hemodynamics partly depends on an increased sympathetic drive. The plasma levels of catecholamines may serve as indicators of sympathetic nerve activity [28]. The plasma levels of catecholamines in the MT group were not lower than those in the UT group, suggesting that metoprolol did not significantly suppress sympathetic outflow from the central nervous system. In contrast, the plasma levels of catecholamines were significantly lower in the DT group than those in the UT group, suggesting that donepezil not only decreased the HR but also suppressed the central sympathetic outflow. Donepezil increases the level of central and peripheral ACh, contributing to the augmented vagal tone and diminished sympathetic outflow [29, 30]. Although extreme sympathetic suppression may collapse the hemodynamics in CHF [31], a moderate suppression of plasma catecholamines may reduce catecholamine cardiotoxicity [32] to improve cardiac function. Furthermore, since ACh reduces HR through a mechanism different from β blockade [33], the additive effects of donepezil and metoprolol on HR reduction in the DMT group are reasonable. During the first week after RMI, the reduction of average daily HR seems to develop more slowly in the DT group than in the MT group (Fig. 2b), which may support the interpretation that the mechanisms underlying the bradycardic effects were different between donepezil and metoprolol.

MBP reached a peak value during week 2 and then tended to decrease in the UT group; these results imply a gradual shift from compensated conditions to decompensated conditions after RMI. The increasing trend of MBP in the DT, MT, and DMT groups may result from preventing cardiac dysfunction and the compensatory mechanism. Bradycardia may play an important role in preventing cardiac dysfunction. A prolonged cardiac cycle enhances and maintains cardiac function by decreasing myocardial oxygen consumption, increasing coronary blood flow, and increasing ventricular filling time [34]. The DMT group showed the most significant bradycardic effect in the present study but did not induce adverse effects, such as severe bradycardia, indicating that donepezil can be used with metoprolol. Therefore, donepezil may serve as an alternative to β-blockers or adjunctive therapy with conventional β-blockers in AMI patients after reperfusion therapy. As the metoprolol monotherapy did not show a significant improvement in cardiac function, whether metoprolol should be added to donepezil in general clinical situations after RMI was inconclusive from the results of the present study and remains to be investigated.

### Effects of donepezil treatment on myocardial salvage

Myocardial salvage of the I/R region is associated with myocardial inflammation during the acute phase after RMI [9, 12]. AMI triggers inflammatory infiltration of neutrophils and macrophages to the damaged heart [35]. A primary function of these phagocytic cells is to remove necrotic and apoptotic cardiac cells and damaged extracellular matrix. These processes are vital for repairing the injured myocardium to preserve myocardial contractility and prevent CHF. The population of different leukocyte subsets dynamically changes during this inflammation. Macrophage polarization first produces the pro-inflammatory M1 phenotype to assist the host against cardiac injury. Subsequently, macrophages are polarized to the M2 phenotype to produce an anti-inflammatory response. As a sign of RMI progress, the inflammatory response was shown to contain a significantly high percentage of M1 cells, which are capable of producing pro-inflammatory responses and pro-inflammatory mediators, such as tumor necrosis factor-α (TNF-α) and inducing myocardial apoptosis. In contrast, there was a significantly low percentage of the M2 phenotype, which plays an important role in damaged cardiomyocyte repair and angiogenesis [36]. The I/R region contained a higher M1/M2 ratio with sustained myocardial apoptosis [24]. It has been demonstrated that inhibition of M1 and stimulation of M2 is a potential therapeutic approach for treating neuroinflammation-related diseases [37].

This study revealed that donepezil monotherapy or combination with metoprolol initiated post-RMI, significantly suppressed neutrophil and total macrophage infiltration in the I/R region 1 week after RMI (compared with the UT group). Furthermore, the DT and DMT groups exhibited reduced production of pro-inflammatory M1 macrophages and increased production of anti-inflammatory M2 macrophages, resulting in a decrease in the M1/M2 ratio and regulation of autophagy [38] in the early stage after RMI. These beneficial effects may contribute to the suppression of local inflammatory expansion and sustained myocardial apoptosis. Thus, early donepezil administration promotes cardiac repair and fibrotic scarring in the acute phase after RMI, thereby increasing myocardial salvage in the I/R region.

### Effects of donepezil treatment on infarct size, cardiac remodeling, and neurohumoral factors

It is a well-established fact that the loss of myocardium in the acute stage after RMI triggers a cascade of intracellular signals, resulting in increased infarct size, scar expansion, and adverse LV remodeling. Infarct size—which is directly affected by myocardial salvage—is a strong predictor of AMI prognosis [5]. Reportedly, every 5% increase in infarct size is associated with a 20% increase in 1-year hospitalization for CHF and 1-year mortality [5, 39, 40]. Despite PCI treatment with drug-eluting stents and early (< 24 h) oral administration of β-blockers, as recommended by clinical practice guidelines, the incidence of CHF after AMI remains high [6]. Here, we showed that infarct size decreased by 18% in the DT group compared with that in the UT group, which corresponded with the suppression of cardiac hypertrophy reflected in the reduced cardiomyocyte cross-sectional area and heart weight. Immunohistological examination confirmed that cardiac fibrosis was reduced, and microvessel densities were increased in the DT group compared with those in the UT group. These benefits may be the structural basis of preserved cardiac function in the DT group. Furthermore, donepezil treatment reduced plasma catecholamine, BNP, and hs-CRP levels, indicating that donepezil suppressed subsequent progression to CHF. However, in this study, early metoprolol monotherapy was found not to exert beneficial effects, except for reducing the hs-CRP levels and HR. These results differed from those observed in rats with CHF following permanent MI, in which metoprolol treatment—initiated 2 weeks after permanent MI—improved cardiac function [23]. Interestingly, the beneficial effects of donepezil were observed in the DMT group; these included the additive effects of HR reduction. These results suggest that the benefits of donepezil in a rat model of RMI are mediated via mechanisms different from those of metoprolol.

### Possible mechanisms of donepezil treatment in RMI

Excessive inflammation and immune dysregulation significantly influence the pathogenesis and development of cardiovascular diseases, such as the worsening of progression of LV remodeling and CHF, which result from cardiac autonomic imbalances [41–43]. This study demonstrated that early (several hours post-RMI) administration of donepezil significantly suppressed cardiac inflammation and prevented cardiac remodeling. Donepezil may similarly exert cardioprotective effects, either in the acute or chronic phase of heart failure. First, by its primary action, donepezil increases the levels of ACh in the brain. Although the precise mechanism remains to be determined, the increased levels of brain ACh may result in increased vagal tone and decreased sympathetic outflow [29], as supported by reduced HR and low levels of plasma catecholamines. Second, ACh is involved in the neural or non-neural cholinergic anti-inflammatory and angiogenic processes. The concept “cholinergic anti-inflammatory pathway” describes a neural mechanism that inhibits pro-inflammatory cytokine release via vagal efferent fibers, which requires signaling through peripheral α7-nicotinic acetylcholine receptors (α7-nAChRs) [43–46]. The involvement of peripheral α7-nAChRs in the treatment mechanism of donepezil has been demonstrated in our previous study [47]. The central-acting AChE inhibitors, which include donepezil and galantamine, have been used to treat Alzheimer’s disease (AD) [48, 49]. Theoretically, these AChE inhibitors may have anti-inflammatory effects. However, the therapeutic action of galantamine is mainly produced by its sensitizing action on nAChRs rather than by general cholinergic enhancement due to cholinesterase inhibition [50]. Whether the cardioprotective effects observed in this study are specific to donepezil or more commonly applicable to central-acting AChE inhibitors awaits further investigations.

Accumulating evidence indicates that inflammation plays a key role in developing the cardiac disease, especially CHF [51, 52]. The initial post-RMI response is characterized by neutrophil and pro-inflammatory M1 macrophage infiltration in the acute inflammatory phase. During the subsequent healing phase, anti-inflammatory M2 macrophages infiltrate the I/R region, contributing to wound repair, neovascularization, restriction of tissue damage, and reparative fibrosis of the infarct zone [41]. Furthermore, the production of pro-inflammatory cytokines, such as TNF-α and interleukin-1β, by M1 cells can stimulate the vagal motor nucleus by stimulating the afferent vagal nerve or via direct action on the central nervous system. As a result, the cholinergic inflammatory response, which is also involved in immune dysregulation post-RMI, is induced [44]. Clinical findings have also demonstrated that peak levels of macrophages negatively correlate with the extent of myocardial salvage in MI patients [35]. Our previous studies indicated the beneficial effects of donepezil on sympathovagal balance, cardiac remodeling, and long-term survival in CHF rats when donepezil treatment was initiated 2 weeks after MI [20, 21, 29]. In the current study, we found that early (several hours post-RMI) administration of donepezil significantly suppressed neutrophil and total macrophage infiltration or reduced the production of pro-inflammatory M1 cells and promoted the infiltration of anti-inflammatory M2 cells in the I/R region by the eighth day. The improvement in myocardial salvage by suppression of apoptosis may have contributed to reduced infarct size and preserved cardiac function (compared with the UT or MT group) by week 10. These results demonstrate that early donepezil intervention may modulate inflammation to promote the healing of the damaged cardiomyocytes in the heart. A recent study reported that intravenous donepezil protected from I/R injury by restoring the balance of mitochondrial dynamics and reducing apoptosis [53]. Our results are in line with these reports. The effects of donepezil were not negatively influenced by the existence of metoprolol in the DMT group, although metoprolol monotherapy failed to exert beneficial effects. The effects of β-blockers are controversial in CHF rats [23, 54]. That being said, there is a possibility that metoprolol exerted an additive anti-inflammatory effect in the DMT group, because hs-CRP was significantly lower in the MT group than in the UT group. The attenuation of serum CRP elevation after AMI by early use of β-blockers has been reported in a clinical study [55]. The beneficial effects of donepezil observed in the DMT group are consistent with the results of a previous study which revealed that electrical vagal nerve stimulation was beneficial in the presence of a β-blocker in CHF rats [23]. Accordingly, our study suggests that the autonomic restoration and cholinergic anti-inflammatory mechanisms of donepezil are involved in the transition from the acute to the chronic phase of cardiac dysfunction following MI.

### Clinical implications

The widespread implementation of PCI improved the survival rate of patients with AMI; however, many patients face the risk of developing CHF. Although β-blockers are the first recommendation for protecting the heart from compensatory sympathetic overdrive, the expected therapeutic effect is not achieved in AMI patients. This study demonstrated first that early donepezil treatment exerts cardioprotective benefits better than metoprolol in rats following RMI. Donepezil not only reduced HR through pharmacological parasympathetic activation but also improved myocardial salvage by cholinergic anti-inflammatory effects, leading to decreased infarct size and prevention of cardiac remodeling. Donepezil, as a central-acting AChE inhibitor, has been used to treat AD. In the clinical studies, the treatment of AD patients with AChE inhibitors significantly lowered the risk of MI and cardiovascular mortality, and new-onset CHF [48, 49]. Furthermore, the effects of donepezil were not affected by the background of the metoprolol in the DMT group. Research data are still lacking to support donepezil as an alternative for β-blockers in patients who are easily decompensated by β-blockers, and future studies are warranted to evaluate donepezil, either alone or in combination with β-blockers, in clinical trials.

## Limitations

First, we focused on cardiac function in the present study and did not examine blood data relating to liver and kidney functions. Although none of the groups showed apparent behavioral anomalies, neurological tests were not performed. Further studies are needed to clarify these points. Second, we used a rat model of RMI-induced CHF to recapitulate the precarious clinical conditions of hospitalized patients with AMI. However, the experimental animals were young, possibly with preserved autonomic function, and might have been more responsive to various therapeutic interventions. Many AMI patients are middle- to old-aged and may have a limited capacity to respond to this novel treatment. Third, because the drugs were dissolved in drinking water, the daily dose was dispersed in each drink volume. Thus, drug plasma levels may have multiple (daily intake frequency) low surges rather than several high peaks (drug administration frequency). Whether the daily intake frequency of the drug influences the outcome of donepezil for RMI may be necessary for future study. Finally, because clinical trials usually involve patients with various backgrounds in pharmacological treatment, it is difficult to determine the efficacy of monotherapy in that population. This may be an important factor to consider when translating the outcomes of this basic study to clinical practice.

## Conclusions

This study first showed that early donepezil monotherapy or donepezil combined with metoprolol significantly increased myocardial salvage through anti-inflammatory mechanisms, as evidenced by reduced macrophage infiltration, improved M1/M2 balance, and decreased myocardial apoptosis. In this rat model of RMI, donepezil reduced myocardial infarct size, prevented cardiac remodeling, and attenuated neurohumoral activation, while metoprolol monotherapy did not produce these benefits. We suggest that donepezil is a potential candidate for adjunct therapy to PCI in patients with AMI.

## Data Availability

The data sets used and/or analyzed during the current study are available from the corresponding author on reasonable request.
